# Clinical, Sonographic, and Hysteroscopic Features of Endometrial Carcinoma Diagnosed after Hysterectomy in Patients with a Preoperative Diagnosis of Atypical Hyperplasia: A Single-Center Retrospective Study

**DOI:** 10.3390/diagnostics12123029

**Published:** 2022-12-02

**Authors:** Luca Pace, Silvia Actis, Matteo Mancarella, Lorenzo Novara, Luca Mariani, Gaetano Perrini, Francesca Govone, Alessandra Testi, Paola Campisi, Annamaria Ferrero, Nicoletta Biglia

**Affiliations:** 1 Gynecology and Obstetrics Unit, Umberto I Hospital, Department of Surgical Sciences, University of Turin, Largo Turati 62, 10128 Turin, Italy; 2 Gynecology and Obstetrics Unit, Umberto I Hospital, Largo Turati 62, 10128 Turin, Italy; 3 Anatomic Pathology Unit, Umberto I Hospital, Largo Turati 62, 10128 Turin, Italy

**Keywords:** atypical endometrial hyperplasia, endometrial cancer, hysteroscopy, transvaginal ultrasound

## Abstract

Background: atypical endometrial hyperplasia (AEH) is a precancerous condition implying a high risk of concurrent endometrial cancer (EC), which might be occult and only diagnosed at postoperative histopathological examination after hysterectomy. Our study aimed to investigate potential differences in preoperative clinical, sonographic, and hysteroscopic characteristics in patients with AEH and postoperative diagnosis of EC. Methods: a retrospective single-center study was carried out on a case series of 80 women with AEH undergoing diagnostic workup, including ultrasonography and hysteroscopy, with subsequent hysterectomy. Women with AEH confirmed at the histopathological examination were compared with patients with a postoperative diagnosis of EC. Results: in our population, EC was diagnosed in 53 women, whereas the preoperative diagnosis of AEH was confirmed in 27 cases. At ultrasonography, women with occult EC showed greater endometrial thickness (20.3 mm vs. 10.3 mm, *p* 0.001) and size of the endocavitary lesion (maximum diameter 25.2 mm vs. 10.6 mm, *p* 0.001), and a higher prevalence of irregular endometrial-myometrial junction (40.5% vs. 6.7%, *p* 0.022) and endouterine vascularization at color Doppler (64.2% vs. 34.6%, *p* 0.017). At hysteroscopy, patients with occult EC showed a higher prevalence of necrosis (44.2% vs. 4.2%, *p* 0.001) and atypical vessels (70.6% vs. 33.3%, *p* 0.003), whereas true AEH mainly presented as a protruding intracavitary lesion (77.8% vs. 50.9%, *p* 0.029). In EC, subjective assessment by the operator was more frequently indicative of cancer (80.0% vs. 12.5%). No difference was found for clinical variables. Conclusions: occult EC in AEH may exhibit some differences in ultrasonographic and hysteroscopic patterns of presentation compared with real AEH, which could prompt a more significant suspect for the possible presence of concurrent EC at preoperative diagnostic workup.

## 1. Introduction

Endometrial hyperplasia (EH) is defined as an irregular proliferation of the endometrial glands leading to an increase in the gland-to-stroma ratio in contrast to proliferative endometrium.

The histological classification of EH has undergone numerous changes over the years, reflecting its diagnostic complexity and making it difficult to compare studies performed with different classifications. In 2014, the World Health Organization (WHO) suggested a dichotomous classification of EH [1], which was accepted by the International Society of Gynecological Pathologists, to reduce the multitude of terms used worldwide. EH has been divided into two groups: non-atypical EH/benign hyperplasia and atypical EH (AEH) or Endometrial Intraepithelial Neoplasia (EIN) according to the presence or absence of atypical cytological features. AEH is a premalignant lesion, with an approximately 30% risk of progression to endometrial carcinoma (EC) [2,3]. The prevalence of concurrent occult EC in patients diagnosed with AEH undergoing hysterectomy approaches 43% [4]. EC is the most common gynecologic cancer in women; it is a more common disease among postmenopausal women, but in the last few years, there has been a rise in the number of EC among premenopausal women [5]. EC is more prevalent in high/intermediate-developed countries [6]. 

Preoperative diagnostics play a central role in defining the correct treatment course of action and surgical approach of AEH and EC. For instance, although lymph node evaluation remains crucial in the surgical management of endometrial carcinoma, there remain no clear consensus guidelines regarding nodal evaluation in patients with AEH.

Pre-operative identification of factors that may help to stratify a patient’s risk of concurrent EC is mandatory to reduce the risk of over- or under-treatment.

Our study aims at evaluating the presence of pre-operative clinical, ultrasonographic, hysteroscopic, and anatomopathological features in patients with a hysteroscopic diagnosis of AEH and a postoperative diagnosis of EC. 

## 2. Materials and Methods

The study was retrospectively conducted on patients who underwent hysterectomy for AEH at the Obstetrics and Gynecology University Department of Mauriziano Umberto I Hospital in Turin from January 2015 to September 2022.

Data were retrieved through a retrospective review of hospital medical records.

The inclusion criteria were women diagnosed with AEH on hysteroscopic endometrial biopsy with a subsequent total hysterectomy and bilateral salpingo-oophorectomy and histopathological examination of the uterus.

The exclusion criteria were the absence of any available report of preoperative ultrasound examination and/or endometrial biopsy, and women who were conservatively managed and/or received medical therapy before surgery.

All patients were referred to our center for diagnostic evaluation, including transvaginal ultrasound (TVUS) and hysteroscopy for abnormal uterine bleeding or after the finding of sonographic anomalies of the endometrium during a routine scan at outpatient clinics. Diagnostic protocol at our center routinely included:

- An interview with the patient to collect anamnestic and clinical data.
- A TVUS performed by an expert highly-trained sonographer (L.M.) with an Affiniti 70 ultrasound machine (Philips, Amsterdam, The Netherlands, 2013) equipped either with a C10-3v Endocavitary Probe with a 3.0–10.0 MHz frequency range; all examinations were performed according to the recommendations of the main international guidelines [7,8].
- A hysteroscopy was performed in an outpatient setting by two highly trained expert operators (A.F. and G.P.) with an endometrial biopsy. All the procedures included vaginoscopy, distension of the uterine cavity with normal saline, diagnostic evaluation of the cervical canal and uterine cavity with visualization of tubal ostia, and targeted biopsy on any suspicious area of the endometrium using a BETTOCCHI^®^ Hysteroscope equipped with bipolar electrode systems [9]. Diagnosis of AEH was made on endometrial specimens according to WHO 2014 criteria [1].

TVUS was always performed at our center before the hysteroscopy assessment. 

After the diagnosis, all the patients included in the study underwent a total hysterectomy and bilateral salpingo-oophorectomy according to the management suggested by the main international guidelines [10,11]. Histopathological examination of the uterus was obtained, either confirming AEH or revealing EC.

Data were retrospectively collected about:

- The anamnestic features, including age, body mass index (BMI), parity, menopausal status, the prevalence of diabetes and hypertension, use of hormone replacement therapy or tamoxifen, and symptoms;
- The ultrasound characteristics regarding endometrial thickness and echogenicity, endometrial–myometrial junction, presence of intracavitary fluid, vascularization at color Doppler (CD) study, size and appearance of the lesion, posterior sliding sign, uterine volume calculated by the formula ellipsoid volume [12], and presence of leiomyomas;
- The hysteroscopic reports about the appearance of the lesion (protruding into the uterine cavity vs. superficial anomaly of the endometrium), presence of necrosis or atypical vascular pattern, subjective assessment indicative of carcinoma by the operator, and visualization of tubal ostia;
- The histopathological reports on the endometrial biopsy regarding the presence of endometrial intraepithelial neoplasia, multiple foci of hyperplasia, and endometrial polyp with AEH arising on its surface, and the number of specimens retrieved by the hysteroscopy operator;
- Histopathological reports on the uterus, most notably the presence of endometrial carcinoma and its features according to WHO 2014 classification [13].

### Statistical Analysis 

The study population was divided into two groups according to the presence or absence of EC at the histopathological examination of the uterus after hysterectomy. The two groups were compared for the variables collected to evaluate potential differences in preoperative features. 

Continuous variables were expressed as mean ± standard error (SE), and categorical variables were expressed as *n* (%). Univariate analysis was performed for continuous variables with a two-tailed *t*-test for independent samples with unequal variances, and categorical variables with a Fisher’s test after checking with the Kolmogorov–Smirnov test that the distribution of our samples did not differ from the normal one. A difference was considered statistically significant when it was associated with a two-tailed *p* < 0.05. 

Statistical analyses were performed using SPSS 22.0 (Statistical Package for the Social Sciences) software (IBM Corp. Released 2013. IBM SPSS Statistics for Windows, Version 22.0. Armonk, NY, USA: IBM Corp.).

## 3. Results

The archiving software (Winsapp vers. 3) of the Pathology Department of Mauriziano Umberto I Hospital was used for patient selection.

Through a search with the query “endometrial hyperplasia” of patients undergoing hysteroscopy, 492 patients with a diagnosis of EH at biopsy were identified.

Of these, 317 were excluded given the diagnosis of EH without atypia. Of the remaining 178, 54 were excluded because they underwent surgery at another center. An additional 32 patients were excluded because a qualitatively inferior and different ultrasound was used compared with the Philips Affiniti 70 model. Two were excluded because of synchronous diagnosis of endometrioid adenocarcinoma of the ovary and inability to establish with certainty the origin of the primary lesion. For seven patients, anamnestic data (abnormal uterine bleeding [AUB], BMI, and comorbidities) could not be found and it was, therefore, decided not to include them in the case series. Our study population included 80 women who were diagnosed with AEH from January 2015 to September 2022 at our center and underwent surgery. Table 1 summarizes the characteristics of this population: most women were post-menopausal (87.0%) with a mean age of 64.9 years; a high prevalence of obesity (45.1%) was seen with a mean BMI 30.7 kg/m^2^; and in 67.5% of cases, AUB was reported and prompted diagnostic evaluation. Among the tests performed during the work-up, TVUS highlighted a high prevalence of endometrial thickening as sonographic presentation, with a mean thickness of 16.4 mm which is far above the high-risk cut-off suggested by the literature [7].

**Figure 1:**
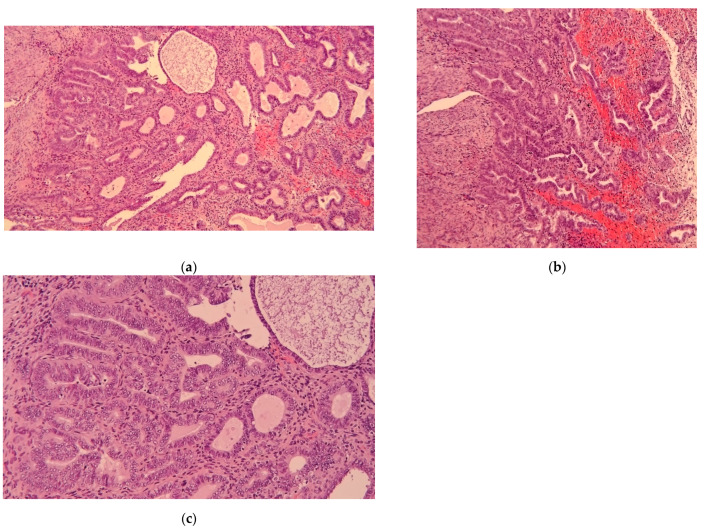
Histopathological images of atypical endometrial hyperplasia (a–c).

At the histopathological examination of the uterus after surgery, EC was revealed in 53 women, whereas the preoperative diagnosis of EAH was confirmed in 27 patients (Figure 1). 

The cases of malignancy were all represented by EC, with coexistent hyperplasia confirmed in 29 out of 53 women (54.7%). Twenty-eight EC cases were histological grade 1 (52.8%), twenty-three cases were classified as grade 2 (43.4%), two cases as grade 3 (3.8%), and lymphovascular invasion was reported in fifteen patients (28.3%). The endometrial invasion was detected in 44 (83%) of the 53 EC. Most EC patients (34 out of 53 women, 66.0%) were classified as stage Ia according to FIGO classification [14]. 

The group of patients with a postoperative diagnosis of EC was compared with the group of women for whom the diagnosis of AEH was confirmed to analyze potential differences in the variables relating to preoperative presentation.

Table 2 shows the results regarding the anamnestic features of the two groups, which appeared to be similar without any statistically significant difference, although patients with EC were on average older (*p* = 0.09).

In Table 3, the main sonographic characteristics of the two groups are shown, highlighting significantly greater endometrial thickness and size of the lesion measured at TVUS for the women with EC. This difference is also relevant in absolute terms, with measures that are on average double the ones reported in AEH patients (notably, 10.3 vs. 20.3 mm for endometrial thickness). Among the other variables, cases of EC showed a significantly higher proportion of irregularity in the appearance of the endometrial–myometrial junction and the presence of endometrial vascularization, expressed by a color score of 2 or higher in the Doppler study.

In Table 4 and Table 5, findings at hysteroscopy and histopathological examination of endometrial biopsies are shown: among women with EC, a significantly higher prevalence of necrosis (44.2%) and atypical vascularization (70.6%) was reported. In about half of the cases, a surface or nodular growth was described for the lesion. On the contrary, in patients with AEH, the most common presentation was a polypoid lesion protruding into the uterine cavity (77.8%), with a frequent histopathological report of atypical cells in the context of an endometrial polyp (73.1%). It is noteworthy that in 80.0% of cases of endometrial carcinoma, a subjective assessment of malignancy was provided by the operator performing hysteroscopy, whereas this evaluation was reported just in 12.5% of cases of hyperplasia.

## 4. Discussion

The present study analyzed several pre-operative factors, including patient characteristics and ultrasonographic, hysteroscopic, and anatomopathological features in patients pre-operatively diagnosed with AEH.

No statistically significant factor suggestive of concomitant EC could be identified regarding the anamnestic data analyzed. Obesity, diabetes, and hypertension were found to be similarly prevalent in both groups under analysis. This is in agreement with the literature, where the above risk factors are common in both diseases, and no medical comorbidities appear to be associated with concurrent EC in patients pre-operatively diagnosed with AEH [3,15]. 

In the present study, women with EC were on average of older age compared with real AEH, although no statistically significant difference was detected (*p* = 0.09). In the literature, older age seems predictive of concurrent EC at the time of hysterectomy for AEH [3,15]. The non-significance of the result in our study could be related to the low sample size.

Among ultrasonographic features, it appears that a thickened endometrial stripe, a greater diameter of the lesion, an interrupted endometrial–myometrial junction, and a high vascular density at CD was associated with increased odds of EC. 

**Figure 2:**
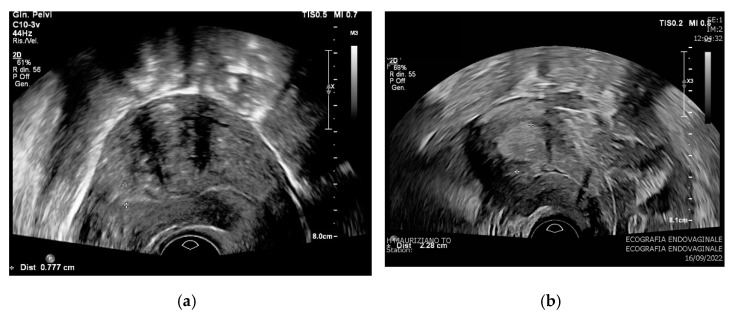
Transvaginal ultrasound (TVUS), endometrial thickness of two patients with a preoperative di-agnosis of atypical endometrial hyperplasia (AEH). (a) TVUS: 7.7 mm of endometrial thickness with a posterior leiomyoma of the uterus, postoperative diagnosis of AEH. (b) TVUS: 22.8 mm of en-dometrial thickness, postoperative diagnosis of endometrial cancer pT1a G2.

Results on endometrial thickness are consistent with prior data from Vetter et al. [3] on a retrospective case series of 169 patients, and from Abt et al. [16] on 378 patients. Both retrospective studies demonstrated that among patients with a preoperative diagnosis of AEH, those with preoperative endometrial stripe ≥ 20 mm were more likely to have concurrent EC. According to a prospective study on 2216 patients with AUB by the International Endometrial Tumor Analysis (IETA), endometrial thickness predictive for AEH is attested at 10.1 mm, while a mean endometrial thickness of 16 mm looks predictive for the EC [17]. The relevance and reproducibility of different studies of this finding should be applied in clinical practice by suggesting that endometrial thickness might be used as one preoperative determinant (Figure 2).

In our study, a greater ultrasonography diameter of the lesion appears to be strongly correlated with the presence of occult EC. This finding is not well investigated in the literature. A retrospective study on 250 patients which analyzed the diagnostic value of endometrial volume under 3D ultrasound acquisition in endometrial lesions demonstrated that the endometrial volume was bigger in the EC group [18]. 

Regarding the ultrasound assessment of the vascularization of the lesion, although this is a remarkably operator-dependent finding, it has been reported in the literature that flow characteristics such as resistance (RI), pulsatility (PI), and peak systolic velocity (PSV) can also help in the differential diagnosis [19].

In the present study, a significant difference was reported for the presence of intracavitary vascularization in the Doppler study, since 64.2% of cases of EC were described as color score 2 or higher at ultrasonography. This result is consistent with the prospective study by Van Den Bosh [20] where a highly vascularized pattern of presentation with a color score of 3 or 4 at CD has been attributed to 65% of EC. 

In the prospective study by Van Den Bosh on 2216 patients, a regular endometrial–myometrial junction at ultrasonography is reported in 65% of AEH, very similar compared with EC in which endometrial–myometrial junction is described as irregular in 42% of cases [20]. In our study, 93.3% of AEH showed regular endometrial–myometrial junction, while only one case (6.3%) had an altered endometrial–myometrial junction. The regular endometrial–myometrial junction at ultrasonography appears in a much lower percentage of EC (40%), in which altered junction was described as irregular in 60% of cases. This result can be analyzed considering the postoperative histologic results; in fact, in our case series, 83% of EC showed endometrial invasion. This data is not available in the previously mentioned study, so we cannot assess inhomogeneity in the case series. Furthermore, this variable is an extremely subjective, highly operator-dependent assessment.

**Figure 3:**
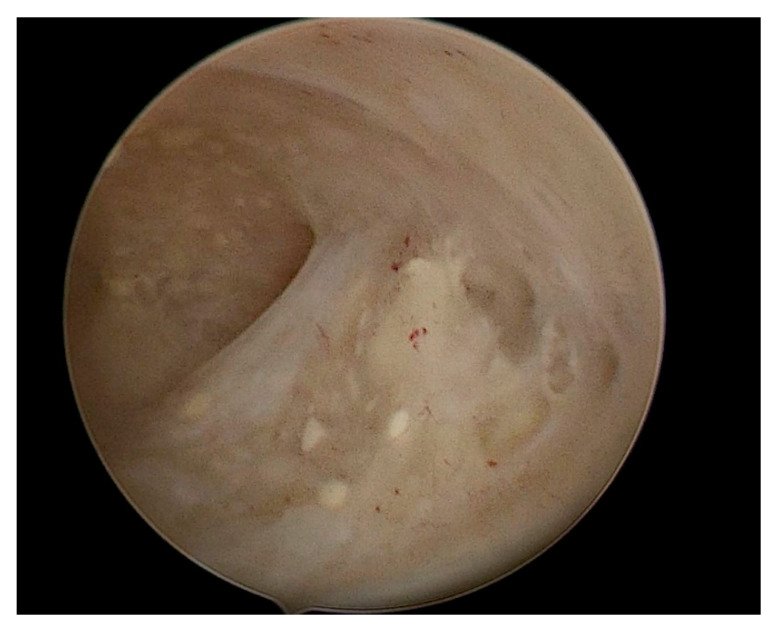
Hysteroscopic image of atypical endometrial hyperplasia (AEH).

Hysteroscopy is considered the gold standard to diagnose endometrial lesions that are clinically or sonographically suspected. Hysteroscopy is a sensitive and specific method to identify coexisting endometrial carcinoma in patients with an AEH diagnosis [21]. Standard hysteroscopy has better results than curette for aspirated endometrial sampling, such as Vabra sampling, which often fails to correctly diagnose endometrial polyps, as the samples have often insufficient endometrial mucosa [22]. That is, the visual assessment of the endometrial cavity reduces blind sampling. Even other poor sensitivity endometrial sampling techniques, such as dilation and curettage, cannot be considered reliable. One of the main advantages of hysteroscopy is the possibility to have a subjective evaluation of the endometrial pattern [23]. It is, therefore, necessary to perform a visual hysteroscopy, as a direct view of the lesion and its relationship to the uterine cavity is necessary for proper assessment (Figure 3).

As for the hysteroscopic phase of preoperative diagnostics in our case series, the presence of necrosis and an atypical vascularization proved to be strongly indicative of EC. 

Necrosis at hysteroscopic evaluation in our study has been much more frequently detected in occult EC than in AEH at postoperative assessment (44.2% vs. 4.2%). This is consistent with the literature where necrosis has been included in many hysteroscopic scores for the diagnosis of suspected EC [23].

**Figure 4:**
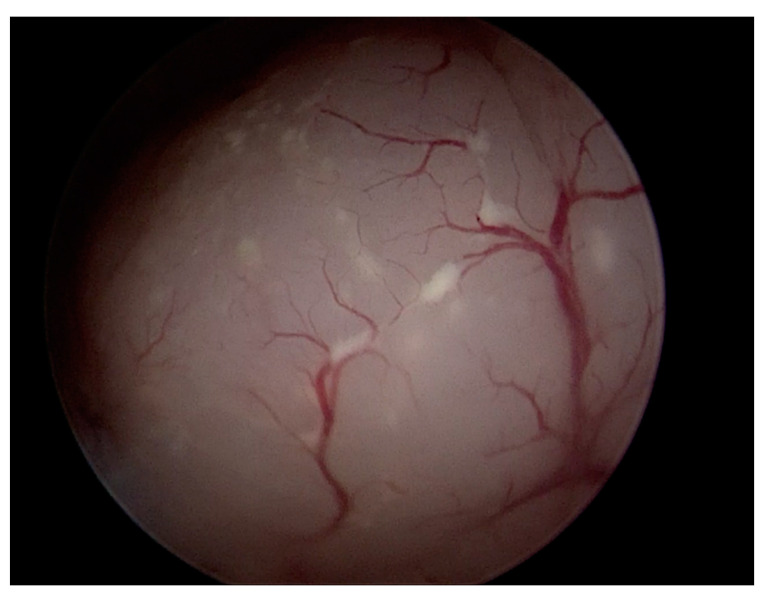
Atypical vascularization at hysteroscopic evaluation.

Atypical vascularization (Figure 4) in our case series, was more frequently found in the case of occult EC (70.6%) compared with AEH (33.3%). Atypical vascularization usually includes the finding of abnormal vessel sprouts, tortuous vessels, vessel loops, branching with angles over 90°, narrowing of vessels, a disorganized network, and an overall irregular distribution with an area with dense vessels, varying with an area without vessels [23]. The abnormal vascularization has been reported to be suggestive of malignant neoplastic lesions of the endometrium, but this finding appears to have been derived from large retrospective cohorts and not from randomized controlled trials [24]. However, a simple increased vascular density must be combined with other parameters in the diagnosis of cancer [25].

The finding of a protruding intracavitary lesion, on the other hand, seems to be more frequent in the case of AEH. This result is consistent with evidence from the literature in which it appears that the hysteroscopic finding of a polyp only rarely correlates with the presence of hyperplasia (about 2% of the cases), and subsequently of a cancerous polyp [22].

The sensitivity and specificity of hysteroscopic subjective assessment in determining the risk of adenocarcinoma have been investigated in numerous studies. The major limitation of this parameter is that it is the result of the subjective evaluation of numerous parameters that are not strictly determined. As a result, hysteroscopic subjective assessment emerges with a wide heterogeneity among different studies. Despite this point, subjective assessment is a valuable tool in the hands of an experienced clinician [15,26,27]. In our case series, subjective assessment ensured superior performance to that found in the literature, with 80% of EC correctly identified by the expert clinician’s report as high-risk lesions. 

To standardize the subjective assessment reports, a structured hysteroscopic score based on lesion surface, necrosis, and vessels has been suggested [23]. Considering the relevance of subjective assessment in the diagnostic procedure of AEH, the definition of standardized and shared criteria for use by experienced operators appears to be a necessary development to improve the diagnostic definition of these lesions.

One of the most controversial issues in the field of endometrial carcinoma is the selection of patients for lymph node staging to avert the risk of understaging. In this regard, preoperative diagnosis of adenocarcinoma is crucial in establishing the correct diagnostic and therapeutic course. Several studies have demonstrated that routine sentinel lymph node biopsy (SLNB) in all patients with AEH has limited benefit and is not cost-effective [28,29,30] given the high prevalence of low-grade and early-stage disease in this category. For AEH and early-stage low-grade EC, a comprehensive surgical staging with lymph node assessment via lymphadenectomy or SLNB would result in overtreatment [31]. Yet, 12% of patients with a pre-operative misdiagnosis of AEH show post-operative histology of EC at a more advanced stage or mid- to high grade. This latter population might benefit from lymph node assessment to guide adjuvant treatment [3]. 

In our case series, almost 34% of the EC had FIGO stage greater than or equal to IB (20.4% of all lesions), and 23% showed lymphovascular invasion. 

The histological features of EC in patients with a previous diagnosis of AEH are remarkably heterogeneous in the literature. Myometrial invasion varies from 30 to 90% depending on the case series, while about 10% of cases show lymphovascular invasion [4,26]. 

This finding underscores the complexity of the histologic evaluation of hysteroscopic biopsy specimens and the need for accurate ultrasound examination by an experienced operator.

A strength of this study is the fact that demographic, anamnestic, hysteroscopic, and ultrasonographic parameters were evaluated in the same group of patients. Furthermore, the case series presented in our study is one of the largest in the literature to date analyzing all of the above parameters together in a single case series. 

The major limitations of this study are the pure retrospective design, which makes it impossible to exclude possible confounding factors, and the fact that some of the variables are strongly based on subjective assessment. Subjective assessment is by its nature operator-dependent and directly influenced by the experience and skills of the operator. As a result, the ultrasound and hysteroscopic evidence of the present study may not widely apply to all centers and may not be universally generalizable. In addition to this, the small sample size may not have allowed additional potentially clinically relevant differences to be identified. Statistical power was not calculated.

Some future insights for improving the preoperative diagnostic definition of AEH can be identified. In any patient with preoperatively diagnosed AEH, the diagnostic evaluation should include both ultrasound and visual hysteroscopy performed by experienced clinicians. To make hysteroscopic parameters more reproducible and reduce the subjectivity of the assessment as much as possible, a consensus between expert operators to define high-risk hysteroscopic characteristics would be necessary. Integration into the diagnostic pathway of a comprehensive score, including both hysteroscopic and sonographic features, may be evaluated in the future. Ultimately, to ensure the best clinical management for high-risk patients with EC-suggestive criteria despite a preoperative diagnosis of AEH, centralized management to specialized EC centers might be suggested. 

## 5. Conclusions

The importance of a detailed preoperative diagnosis of AEH and the difficulty in defining AEH on hysteroscopic biopsy dictate careful evaluation of features associated with the finding of AEH. Occult EC cases diagnosed after hysterectomy for AEH may have some differences at preoperative diagnostic workup compared with confirmed AEH cases. In our study, the endometrial thickness and other ultrasonographic features, such as thickened endometrial stripe, a greater diameter of the lesion, an interrupted endometrial–myometrial junction, and a high vascular density at CD, along with the subjective hysteroscopic assessment by experienced clinicians are elements that can suggest the presence of occult EC in patients with a preoperative histologic diagnosis of AEH.

The results of our study should be prospectively verified on larger and prospective case series. A multicenter prospective study should be conducted based on the prospective use of an inclusive score of standardized clinical, hysteroscopic, and ultrasound features in the preoperative diagnostic pathway. This is also to select a population of patients with a pre-operative misdiagnosis of AEH who might benefit from lymph node assessment to guide adjuvant treatment.

Patients with a pre-operative misdiagnosis of AEH show post-operative histology of EC at a more advanced stage or mid- to high grade. This latter population might benefit from lymph node assessment to guide adjuvant treatment.

## Figures and Tables

**Table 1:** Characteristics of the study population.

Category	Characteristics	
**Clinical features**	Age at diagnosis (years) *	64.9 ± 1.1
	BMI (kg/m^2^) *	30.7 ± 1.0
	Obesity (%)	32 (45.1)
	Diabetes (%)	16 (21.6)
	Hypertension (%)	40 (54.1)
	Number of VD *	1.2 ± 0.1
	Post-menopausal status (%)	67 (87.0)
	Time between menopause and diagnosis (years)	14.7 ± 1.1
	Use of HRT (%)	0 (0)
	Use of tamoxifene (%)	5 (6.9)
	Presence of AUB (%)	52 (67.5)
**Ultrasonography features**	Endometrial thickness (mm) *	16.3 ± 1.7
	Non-uniform endometrial echogenicity (%)	10 (12.5)
	Irregular endometrial–myometrial junction (%)	18 (31.6)
	Intracavitary fluid (%)	6 (8.5)
	Intracavitary vascularization at CD (%)	43 (54.4)
	Focal endometrial lesion (%)	24 (37.5)
	Maximum diameter of the lesion (ml) *	22.0 ± 2.5
	Volume of the uterus (cm^3^) *	76.4 ± 6.6
	Presence of uterine fibroids (%)	29 (41.4)
**Hysteroscopy features**	Protruding intracavitary lesion (%)	48 (60)
	Necrosis (%)	24 (31.6)
	Atypical vascularization (%)	44 (58.7)
	Visualization of tubal ostia (%)	80 (100)
	Subjective assessment suggesting cancer (%)	43 (58.1)
	EH on endometrial polyps (%)	41 (52.6)
	EIN (%)	6 (7.7)
	Multiple foci of hyperplasia (%)	30 (42.9)
	Number of endometrial biopsies *	1.7 ± 0.07

BMI, body mass index; VD, vaginal delivery; HRT, hormone replacement therapy; CD, color Doppler; AUB, abnormal uterine bleeding; EH, endometrial hyperplasia; EIN, endometrial intraepithelial neoplasia; patients are classified as obese when their body mass index (BMI) is over 30 kg/m^2^. *: data are reported as mean ± standard error.

**Table 2:** Anamnestic features of the study groups.

Variables	Endometrial Hyperplasia (N = 27)	Endometrial Carcinoma (N = 53)	*p* ^§^
Age at diagnosis (years) *	62.3 ± 1.8	66.2 ± 1.4	0.09
BMI (kg/m^2^) *	29.3 ± 1.5	31.4 ± 1.2	0.29
**Obesity (%)**	11 (47.8)	21 (43.8)	0.80
**Diabetes (%)**	5 (20.8)	11 (22.0)	0.91
**Hypertension (%)**	11 (45.8)	29 (58)	0.46
Number of VD *	1.1 ± 0.2	1.3 ± 0.2	0.42
**Post-menopausal status (%)**	23 (88.5)	44 (86.3)	0.79
Time between menopause and diagnosis (years) *	12.6 ± 1.9	15.7 ± 1.4	0.19
**Use of HRT (%)**	0 (0)	0 (0)	-
**Use of tamoxifene (%)**	1 (4.0)	4 (8.5)	0.65
**Presence of AUB (%)**	15 (57.7)	37 (72.5)	0.21

BMI, body mass index; VD, vaginal delivery; HRT, hormone replacement therapy; AUB, abnormal uterine bleeding; patients are classified as obese when their body mass index (BMI) is over 30 kg/m^2^. *: data are reported as mean ± standard error. ^§^: analysis was carried out with a two-tailed *t*-test for independent samples with unequal variances for continuous variables, and with Fisher’s test for categorical variables.

**Table 3:** Ultrasound features of the endometrial lesions in the two groups.

Variables	Endometrial Hyperplasia (N = 27)	Endometrial Carcinoma (N = 53)	*p* ^§^
Endometrial thickness (mm) *	10.3 ± 1.3	20.3 ± 2.4	**0.001**
**Non-uniform endometrial echogenicity (%)**	2 (7.4)	8 (15.1)	0.48
**Irregular endometrial-myometrial junction (%)**	1 (6.7)	17 (40.5)	**0.022**
**Intracavitary fluid (%)**	2 (8.7)	4 (8.3)	0.96
**Intracavitary vascularization at CD (%)**	9 (34.6)	34 (64.2)	**0.017**
**Focal endometrial lesion (%)**	8 (44.4)	16 (34.8)	0.57
Maximum diameter of the lesion (mm) *	10.6 ± 2.5	25.2 ± 3.0	**0.001**
Volume of the uterus (cm^3^) *	78.5 ± 10.4	75.6 ± 8.3	0.83
**Presence of uterine fibroids (%)**	6 (27.3)	23 (47.9)	0.12

CD, color Doppler. *: data are reported as mean ± standard error. ^§^: analysis was carried out with a two-tailed *t*-test for independent samples with unequal variances for continuous variables, and with Fisher’s test for categorical variables.

**Table 4:** Hysteroscopic findings in the two groups.

Variables	Endometrial Hyperplasia (N = 27)	Endometrial Carcinoma (N = 53)	*p* ^§^
**Protruding intracavitary lesion (%)**	21 (77.8)	27 (50.9)	**0.029**
**Necrosis (%)**	1 (4.2)	23 (44.2)	**0.001**
**Atypical vascularization (%)**	8 (33.3)	36 (70.6)	**0.003**
**Visualization of tubal ostia (%)**	27 (100)	53 (100)	**-**
**Subjective assessment suggesting cancer (%)**	3 (12.5)	40 (80.0)	**0.001**

^§^ analysis was carried out with a two-tailed *t*-test for independent samples with unequal variances for continuous variables, and with Fisher’s test for categorical variables.

**Table 5:** Histopathologic pre-operative features of the endometrial lesions in the two groups.

Variables	Endometrial Hyperplasia (N = 27)	Endometrial Carcinoma (N = 53)	*p* ^§^
**EH on endometrial polyp (%)**	19 (73.1)	22 (42.3)	**0.016**
**Multiple foci of hyperplasia (%)**	11 (44.0)	19 (42.2)	0.86
Number of endometrial biopsies *	1.7 ± 0.1	1.7 ± 0.1	0.94

EH, endometrial hyperplasia; EIN, endometrial intraepithelial neoplasia. *: data are reported as mean ± standard error. ^§^: analysis carried out with a two-tailed *t*-test for independent samples with unequal variances for continuous variables, and with Fisher’s test for categorical variables.

## Data Availability

The data presented in this study are available on request from the corresponding author.
